# Association of type 2 diabetes, hypertension, and hyperlipidemia with immune-related adverse events in patients undergoing immune checkpoint inhibitors therapy

**DOI:** 10.3389/fimmu.2025.1472197

**Published:** 2025-03-07

**Authors:** Ruidan Li, Xiaoli Mu, Zheran Liu, Rendong Huang, Xingchen Peng

**Affiliations:** ^1^ Department of Biotherapy, Cancer Center, West China Hospital, Sichuan University, Chengdu, China; ^2^ Hangzhou Linan Guorui Health Industry Investment Co.,Ltd., Hangzhou, China

**Keywords:** immune checkpoint inhibitors, immune-related adverse events, type 2 diabetes, hypertension, hyperlipidemia

## Abstract

**Aims:**

Immune-related adverse events (irAEs) pose a significant challenge to the clinical use of immune checkpoint inhibitors (ICIs) in cancer immunotherapy. This study aims to determine whether comorbid conditions such as type 2 diabetes (T2DM), hypertension, and hyperlipidemia affect the risk of irAEs in cancer patients receiving ICIs treatments.

**Materials and methods:**

We conducted a retrospective analysis of clinical data from 3,489 cancer patients treated with ICIs (anti-PD-1, anti-PD-L1, and anti-CTLA-4) at West China Hospital of Sichuan University from 2017 to 2022. Logistic regression models were used to evaluate the associations between T2DM, hypertension, and hyperlipidemia with irAEs. Subgroup analyses assessed irAEs in patients with and without these comorbidities across different cancer types. Additionally, we explored the associations between comorbidities and irAEs affecting different organs.

**Results:**

The results showed that comorbid T2DM, hypertension, and hyperlipidemia significantly increased the risk of irAEs in all cancer types (T2DM: OR=1.40, 95% CI: 1.12-1.74, p=0.003; hypertension: OR=1.21, 95% CI: 1.00-1.45, p=0.049; hyperlipidemia: OR=1.62, 95% CI: 1.02-2.53, p=0.038). T2DM primarily increased the risk of irAEs in lung cancer patients (OR = 1.50, 95% CI: 1.12-2.01, FDR-adjusted p = 0.036), and all three comorbidities significantly elevated the risk of cardiac irAEs.

**Conclusions:**

Our study is the first to confirm an association between T2DM, hypertension, and hyperlipidemia and the occurrence of irAEs in cancer patients receiving ICIs therapy. This finding highlights the critical need for clinicians to perform comprehensive evaluations of patients’ comorbidities prior to treatment.

## 1 Introduction

Immune checkpoint inhibitors (ICIs) have significantly advanced cancer treatment by generating durable responses in many previously intractable malignant tumors (1). However, immune-related adverse events (irAEs) pose a substantial challenge to the clinical application of ICIs (2). Despite extensive research on the mechanisms of irAEs, there is still a lack of comprehensive analysis regarding the risk factors for irAEs across different patient populations, particularly in those with specific comorbidities (3). This gap hinders our understanding of how irAEs present in various groups, which in turn complicates the effective management of the benefit-risk ratio in ICIs therapy (4).

The prevalence of type 2 diabetes (T2DM), hypertension, and hyperlipidemia is rising annually, leading to an increase in comorbidities among cancer patients (5–8). Previous research has suggested that T2DM and hyperlipidemia may be potential immune-metabolic disorders that affect the activation, proliferation, and mobilization of immune cells (9, 10). Hyperglycemia and dyslipidemia are primary causes of metabolic homeostasis imbalance, which induces metabolic adaptations in immune cells and alters overall immune status. Additionally, studies have shown that hypertension can be a pro-inflammatory stimulus, increasing endothelial expression of cytokines and stimulating inflammation (11). These research findings consistently indicated that T2DM, hypertension, and hyperlipidemia have pro-inflammatory effects, revealing their regulatory roles in the systemic immune environment. This provides a theoretical basis for the association between these comorbidities and an increased risk of irAEs. However, there is currently a lack of clinical evidence to explore the specific associations between the occurrence of irAEs and T2DM, hypertension, and hyperlipidemia in patients undergoing ICIs therapy. Further research is necessary to clarify how these comorbidities affect the risk of irAEs in patients treated with ICIs, thereby providing stronger support for clinical decision-making and personalized treatment.

In this study, we retrospectively analyzed the clinical information of patients treated with ICIs. Our results indicate that the risk of irAEs is significantly increased in cancer patients with T2DM, hypertension, and hyperlipidemia. This finding not only provides new insights into the impact of comorbidities on the risk of irAEs but also lays a foundation for the clinical management of this specific patient population. By identifying high-risk patients, we can offer more targeted monitoring and intervention strategies for physicians, optimizing treatment outcomes and reducing the negative impact of irAEs on patients’ quality of life. Furthermore, the results of this study will encourage future prospective research to further explore the roles of these comorbidities in immunotherapy, ultimately providing scientific evidence to improve the overall prognosis of cancer patients.

## 2 Method

### 2.1 Patient cohort and data collection

We retrospectively collected data from cancer patients who received ICIs (anti-PD-1, anti-PD-L1 and anti-CTLA-4) treatments at West China Hospital of Sichuan University between 2017 and 2022. Demographic and clinical information was obtained from electronic medical records using each patient’s unique hospital registration ID. Collected data included age, gender, cancer type, anti-tumor treatment (ICIs only, ICIs + chemoradiotherapy, ICIs + chemotherapy, ICIs + radiotherapy, and ICIs + targeted therapy), ICIs drugs, specified irAEs, and comorbidities (T2DM, hypertension, and hyperlipidemia).

The inclusion and exclusion criteria were as follows: Inclusion criteria: (1) Diagnosis of a primary solid tumor. (2) History of ICIs treatment. Exclusive criteria: (1) Patients without available information. Finally, A total of 3,489 patients were included in the study.

### 2.2 Comorbidities and irAEs

T2DM (ICD-10 codes: E11), hypertension (ICD-10 codes: I10), and hyperlipidemia (ICD-10 code: E78) were identified according to the 10th revision of the International Classification of Diseases (ICD-10).

Patients who developed at least one irAE during ICIs treatment were classified into the irAEs group. The irAEs were categorized based on the primary system organ affected. Cases involving multiple significantly affected organs were classified as multi-organ involvement.

### 2.3 Statistical analysis

Continuous variables were reported as mean and standard deviation (SD), while categorical data were expressed as counts and respective percentages.

Logistic regression models were used to assess the associations between T2DM, hypertension, and hyperlipidemia with irAEs respectively. Benjamini-Hochberg adjustment for multiple comparisons was performed by applying the ‘p.adjust’ function from the ‘stats’ R package to the test p-values. All analyses were performed using R version 4.3.2. The results were reported as odds ratios (ORs) with 95% confidence intervals (CIs). A two-sided p-value of less than 0.05 was considered statistically significant.

## 3 Results

### 3.1 Patient characteristics

**Figure 1:**
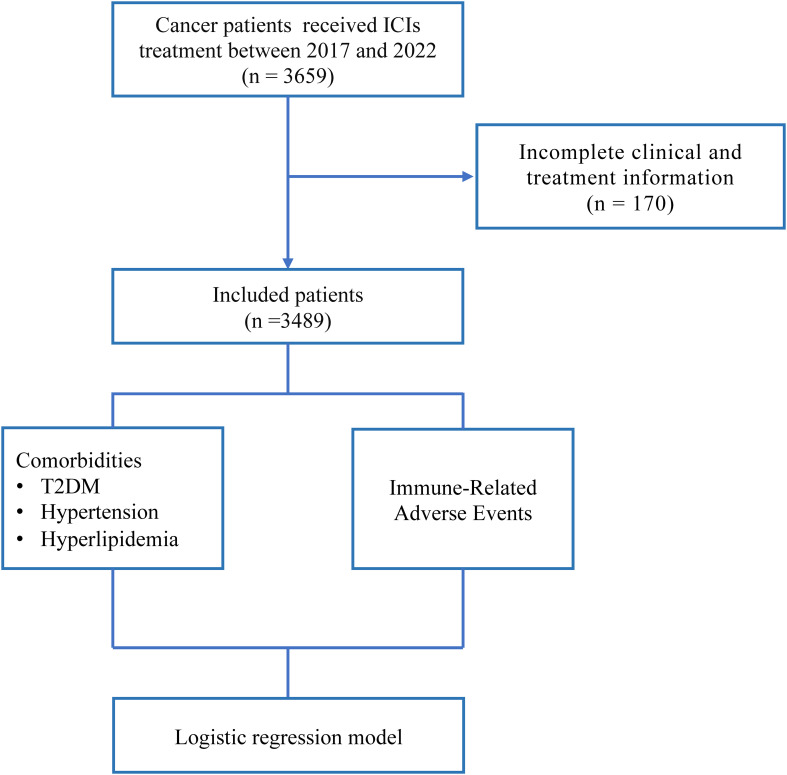
Overview of the design of this study. T2DM, Type 2 Diabetes; ICIs, Immune Checkpoint Inhibitors.

Based on the inclusion and exclusion criteria, 3,489 patients were included in this analysis (**Figure 1**). The characteristics of these patients were presented in **Table 1**. The mean age of the cohort was 59 years (SD = 11.46), with males comprising 75.6% (n = 2,639) of the population. Among all patients, 1,796 (51.5%) had lung cancer, 339 (9.7%) had liver cancer, and other notable tumor types included nasopharyngeal carcinoma (199, 5.7%), gastric cancer (195, 5.6%), head and neck tumors (115, 3.3%), and pancreatic cancer (104, 3%). A majority of the patients underwent combination anti-tumor therapy, including chemoradiotherapy (745, 21.4%), chemotherapy (720, 20.7%), radiotherapy (609, 17.5%), or targeted therapy (157, 4.5%) in addition to ICIs. The most common irAEs were gastrointestinal symptoms (362, 38.6%). Among all cancer patients, 443 (12.7%) had comorbid T2DM, 722 (20.7%) had comorbid hypertension, and 83 (2.4%) had comorbid hyperlipidemia.

**Table 1:** Baseline characteristics of included participant.

Characteristic	Overall (n = 3489)
**Mean (SD) age, year**	59.00 (11.46)
Gender, no (%)	
Female	850 (24.4)
Male	2639 (75.6)
Cancer type, no (%)	
Lung cancer	1796 (51.5)
Esophageal cancer	339 (9.7)
Nasopharyngeal carcinoma	199 (5.7)
Gastric cancer	195 (5.6)
Liver cancer	133 (3.8)
Head and neck cancer	115 (3.3)
Pancreatic cancer	104 (3.0)
Other	608 (17.4)
Drugs, no (%)	
Anti-PD-1	3155 (90.4)
Anti-PD-L1	314 (9.0)
Anti-CTLA-4	20 (0.6)
Therapy, no (%)	
ICIs only	1249 (35.9)
ICIs + chemo+radiotherapy	745 (21.4)
ICIs + chemotherapy	720 (20.7)
ICIs + radiotherapy	609 (17.5)
ICIs + targettherapy	157 (4.5)
Specified irAEs, no (%)	
Gastrointestinal irAEs	362 (38.6)
Pneumonitis	160 (17.1)
Thyroid irAEs	136 (14.5)
Skin irAEs	132 (14.1)
Cardiac irAEs	122 (13.0)
Hepatic irAEs	9 (1.0)
Hematologic irAEs	4 (0.4)
Multi-organ irAEs	5 (0.5)
Other	7 (0.7)
T2DM, no (%)	
No	3046 (87.3)
Yes	443 (12.7)
Hypertension, no (%)	
No	2767 (79.3)
Yes	722 (20.7)
Hyperlipidemia, no (%)	
No	3406 (97.6)
Yes	83 (2.4)

Anti-PD-1, Anti-Programmed Death Receptor 1; Anti-PD-L1, Anti-Programmed Death Ligand 1; Anti-CTLA-4, Anti-Cytotoxic T Lymphocyte-Associated Protein 4; ICIs, Immune Checkpoint Inhibitors; irAEs, Immune-Related Adverse Events; T2DM, Type 2 Diabetes.

### 3.2 Univariate logistic regression analysis of the association between comorbidities and irAEs

In the univariate logistic regression analysis, we examined the associations between T2DM, hypertension, hyperlipidemia, and irAEs. The results indicated that T2DM, hypertension, and hyperlipidemia were all significantly associated with an increased risk of irAEs (**Supplementary Table S1**). Specifically, patients with T2DM had a 40% higher likelihood of experiencing irAEs compared to those without T2DM (OR = 1.40, 95% CI: 1.12 - 1.73, p = 0.002). Additionally, hypertension was significantly associated with a higher likelihood of irAEs compared to those without hypertension (OR = 1.25, 95% CI: 1.04 - 1.50, p = 0.0115), and hyperlipidemia was also significantly linked to an increased risk of irAEs compared to those without hyperlipidemia (OR = 1.64, 95% CI: 1.03 - 2.56, p = 0.031).

### 3.3 Multivariate logistic regression analysis of the association between comorbidities and irAEs

**Figure 2:**
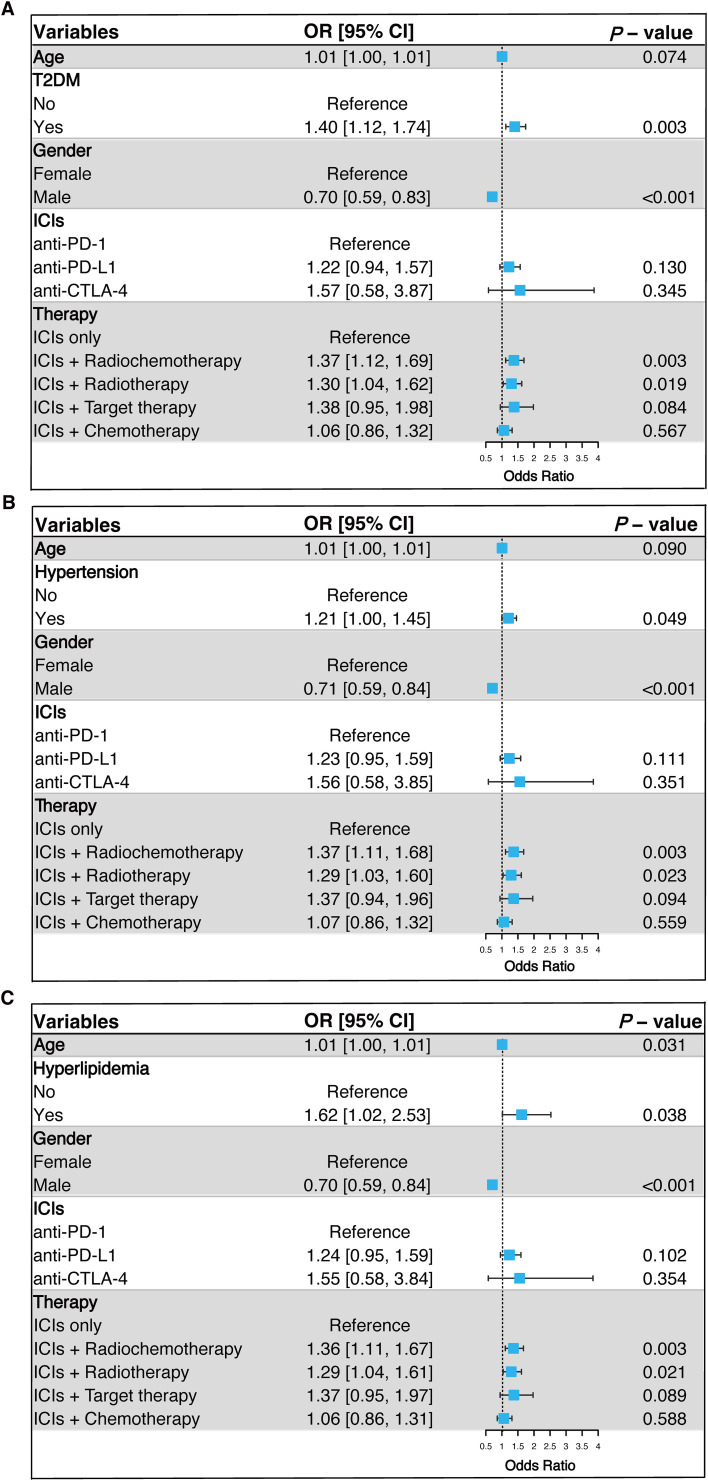
Multivariable logistic regression analysis of the association between T2DM **(A)**, hypertension **(B)**, and hyperlipidemia **(C)** with irAEs. OR, Odds Ratio; CI, Confidence Interval; T2DM, Type 2 Diabetes; ICIs, Immune Checkpoint Inhibitors; Anti-PD-1, Anti-Programmed Death Receptor 1; Anti-PD-L1, Anti-Programmed Death Ligand 1; Anti-CTLA-4, Anti-Cytotoxic T Lymphocyte-Associated Protein 4; irAEs, Immune-Related Adverse Events.

To control for potential confounding variables, we conducted a multivariate logistic regression analysis, including age, gender, ICIs drugs, and anti-tumor treatment as covariates. The results demonstrated that T2DM, hypertension, and hyperlipidemia significantly increased the risk of irAEs. Specifically, T2DM was associated with a 40% higher risk of irAEs (OR = 1.40, 95% CI: 1.12 - 1.74, p = 0.003), hypertension with a 21% higher risk (OR = 1.21, 95% CI: 1.00 - 1.45, p = 0.049), and hyperlipidemia with a 62% higher risk (OR = 1.62, 95% CI: 1.02 - 2.53, p = 0.038). Notably, male patients had a significantly lower risk of developing irAEs compared to female patients (p < 0.001). Additionally, patients receiving concurrent radiotherapy with ICIs exhibited a significantly increased risk of irAEs (**Figure 2**).

### 3.4 The association between comorbidities and irAEs in different cancer types

**Figure 3:**
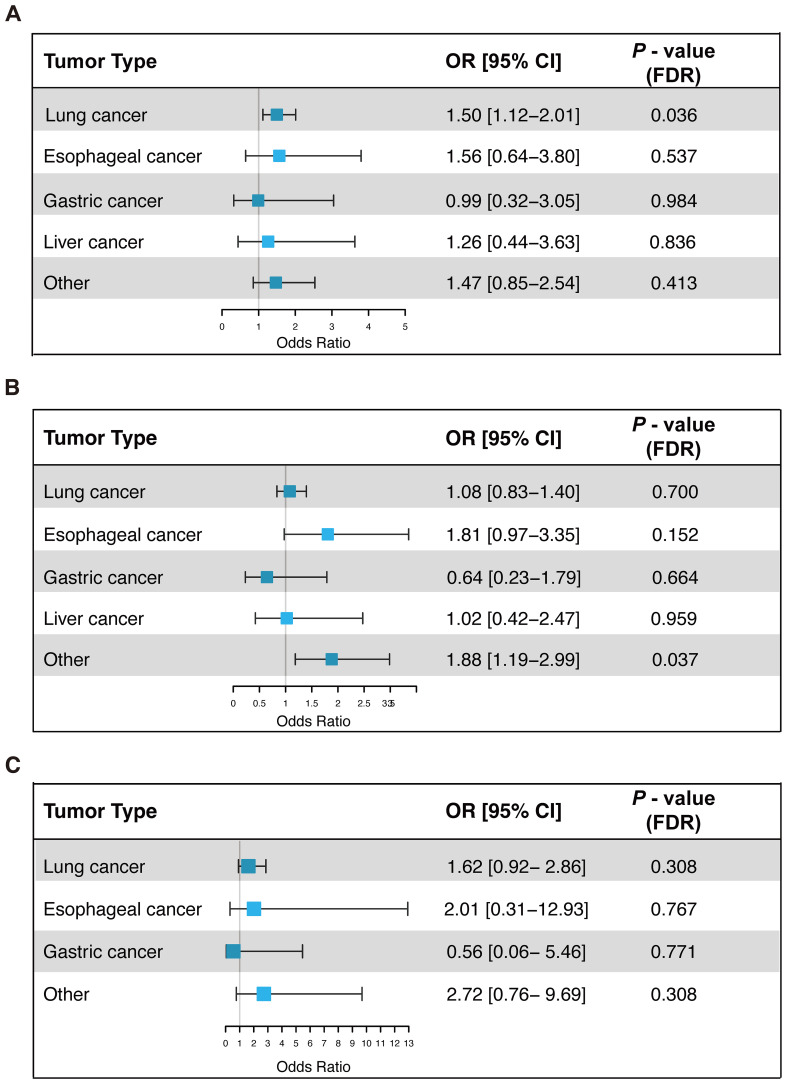
Univariate logistic regression analysis of the association between T2DM **(A)**, hypertension **(B)**, and hyperlipidemia **(C)** with irAEs in different cancer types. OR, Odds Ratio; CI, Confidence Interval; FDR, False Discovery Rate; irAEs, Immune-Related Adverse Events.

To clarify the role of T2DM, hypertension, and hyperlipidemia in the risk of irAEs across different cancer types, subgroup analyses were conducted. Multivariable logistic regression models were employed to adjust for potential confounders, including age, gender, ICIs drugs, and anti-tumor treatments. To minimize the risk of false positives due to multiple comparisons, Benjamini-Hochberg adjustment was applied to the P-values, ensuring the robustness of findings (**Figure 3**). The results indicated that the association between T2DM and increased irAEs risk was primarily driven by lung cancer patients (OR = 1.50, 95% CI: 1.12 - 2.01, FDR-adjusted p = 0.036). The association between hypertension and increased irAEs risk was significant in patients with other cancers (Other cancers: OR = 1.88, 95% CI: 1.19 - 2.99, FDR-adjusted p = 0.037). Although hyperlipidemia was associated with irAEs in the pan-cancer analysis, this association was not statistically significant within individual cancer types, possibly due to smaller sample sizes and heterogeneity among tumor types.

### 3.5 The association between comorbidities and irAEs affected different organs

To better understand the association between comorbidities and organ-specific irAEs, we performed an exploratory analysis (**Table 2**). After adjusting for factors such as age, gender, ICI drugs, and anti-tumor treatments, the results indicated that hypertension was linked to a higher risk of cardiac irAEs (OR = 1.72, 95% CI: 1.12-2.63, FDR-adjusted p = 0.033), but it was associated with a decreased risk of thyroid irAEs (OR = 0.44, 95% CI: 0.25-0.78, FDR-adjusted p = 0.024). Hyperlipidemia also increased the risk of cardiac irAEs (OR = 3.28, 95% CI: 1.47-7.30, FDR-adjusted p = 0.018) in patients treated with ICIs.

**Table 2:** Analysis of the association between comorbidities and irAEs in different organs.

Variables	T2DM		Hypertension		Hyperlipidemia	
	OR [95%CI]	*P* – value (FDR)	OR [95%CI]	*P* – value (FDR)	OR [95%CI]	*P* – value (FDR)
Gastrointestinal irAEs	0.78[0.53, 1.14]	0.413	0.92[0.66, 1.27]	0.600	0.53[0.23, 1.20]	0.214
Pneumonitis	0.95[0.58, 1.54]	0.911	0.84[0.55, 1.30]	0.546	0.16[0.02, 1.18]	0.182
Thyroid irAEs	0.70[0.39, 1.28]	0.414	0.44[0.25, 0.78]	0.024*	0.92[0.31, 2.68]	0.872
Skin irAEs	1.03[0.61, 1.75]	0.911	1.49[0.95, 2.34]	0.131	1.56[0.62, 3.91]	0.432
Cardiac irAEs	1.68[1.05, 2.69]	0.154	1.72[1.12, 2.63]	0.033*	3.28[1.47, 7.30]	0.018*

irAEs, Immune-Related Adverse Events; T2DM, Type 2 Diabetes; OR, Odd Ratio; CI, Confidence Interval; FDR, False Discovery Rate. *P <0.05.

In the preliminary results, we observed that T2DM significantly increased the overall risk of irAEs. However, upon further analysis of the association between T2DM and organ-specific irAEs, none of the associations between T2DM and organ-specific irAEs reached statistical significance (FDR-adjusted p > 0.05). This result may be due to the more stringent significance threshold following the Benjamini-Hochberg adjustment.

## 4 Discussion

In this study, we analyzed clinical data from 3,489 cancer patients treated with ICIs to evaluate the impact of comorbidities—specifically T2DM, hypertension, and hyperlipidemia—on the incidence of irAEs. Our findings revealed a significant association between these comorbidities and an increased risk of irAEs. Specifically, T2DM was linked to a 40% higher risk, hypertension to a 21% higher risk, and hyperlipidemia to a 62% higher risk of developing irAEs. T2DM primarily increased the risk of irAEs in lung cancer patients; meanwhile, hypertension and hyperlipidemia significantly elevated the risk of cardiac irAEs. These findings highlight the critical need to integrate the management of comorbidities into treatment plans for cancer patients receiving ICIs therapy. By identifying these conditions as significant contributors to the risk of irAEs, this study underscores the potential for personalized treatment strategies that optimize the balance between therapeutic efficacy and the minimization of irAEs. Incorporating regular monitoring and proactive management of these comorbidities could not only improve patient outcomes but also enhance the safety and long-term viability of ICIs therapy in diverse cancer populations.

**Figure 4:**
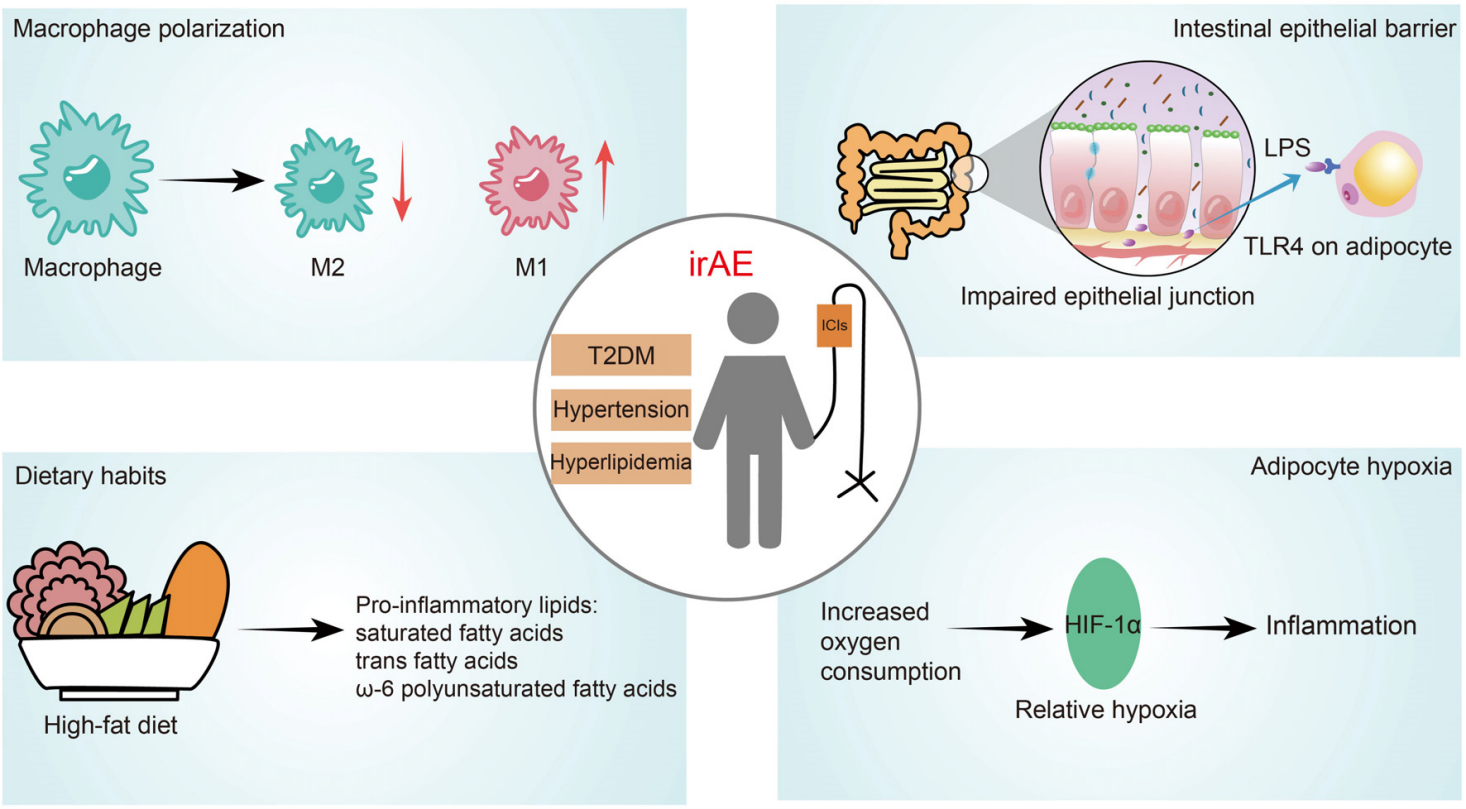
Graphical summary of potential mechanisms for comorbidities and irAEs. irAEs, Immune-Related Adverse Events; LPS, lipopolysaccharides.

irAEs can affect any organ, manifesting as localized signs of systemic immune dysfunction. Therefore, it is crucial in managing irAEs to consider other systemic diseases that may influence overall immune function (12, 13). Research on the association between comorbidities in cancer patients and irAEs is currently limited. Drawing from a large cohort of cancer patients treated with ICIs, our study identified a significant association between the presence of comorbidities, such as T2DM, hypertension, and hyperlipidemia, and an increased risk of developing irAEs. The biological mechanisms underlying these associations likely involve intricate interactions between metabolic pathways and immune responses (14). T2DM, hypertension, and hyperlipidemia often coexist with obesity (15–17). Increasing evidence indicates that obesity can induce chronic low-grade inflammation, activating inflammatory processes during adipose tissue expansion and persistently skewing the immune system towards a pro-inflammatory state (**Figure 4**) (18, 19). A notable feature of obesity-related adipose tissue inflammation is an elevated number of macrophages and an altered ratio of M1 to M2 macrophages, which increases the release of pro-inflammatory factors (15, 20, 21). Obesity also heightens intestinal permeability, elevating circulating levels of lipopolysaccharides (LPS) derived from Gram-negative bacteria in the gut (22, 23). These gut-derived LPS can trigger inflammatory cascades by activating pattern recognition receptors such as Toll-like receptor 4 in adipocytes (24). Additionally, elevated levels of specific lipid species, such as free fatty acids or triglycerides, induced by diet or obesity, can contribute to inflammation (25). Obesity can also induce inflammation through distinct mechanisms such as hypoxia and increased mechanical pressure on adipocytes (19, 26, 27).

Vascular endothelial cells and myocardium are principal targets of pro-inflammatory pathways, which may explain the increased risk of cardiac irAEs associated with hypertension or hyperlipidemia (28–30). Notably, our research indicated a decreased risk of thyroid irAEs in cancer patients with concurrent hypertension, which may be the result of multiple mechanisms regulating immune response, metabolic effects, and drug action. Furthermore, our findings showed that T2DM significantly increased the risk of irAEs in lung cancer patients. Previous literature suggested that compared to non-diabetic patients, intestinal microbial diversity is generally reduced in diabetic patients (31). This reduction in microbial diversity not only disrupts the bidirectional crosstalk of the gut-lung axis, leading to respiratory hypersensitivity and overreaction, but also upregulates genes related to neutrophil and T cell activation, further increasing the risk of irAEs (32).

In this study, we focused on a large cohort of chronic disease patients in China and found that those with T2DM, hypertension, and hyperlipidemia undergoing ICIs therapy face a significantly higher risk of developing irAEs. This discovery enhances our understanding of how these chronic conditions influence irAEs and underscores the need for better prevention and management strategies in clinical practice. By identifying these chronic diseases as independent risk factors for irAEs, clinicians can now conduct more thorough risk assessments before initiating ICIs therapy, opening new opportunities for personalized treatment plans. Early interventions, such as optimizing blood glucose, blood pressure, and lipid levels, can help reduce the likelihood of irAEs and improve patient tolerance to ICIs therapy. Furthermore, the findings underscored the need for more frequent monitoring of chronic disease patients receiving ICIs therapy. Regular follow-up and early detection of potential adverse reactions allow clinicians to manage comorbidities more effectively, reducing the risk of irAEs. This proactive approach not only improves patient outcomes but also enhances the overall safety and effectiveness of ICIs therapy, ultimately maximizing its therapeutic benefits.

Several limitations should be noted. Firstly, the retrospective study design introduces the potential for selection bias and unaccounted confounding factors, such as race, lifestyle habits, and comorbidities, which may have influenced the results. Secondly, the single-center design limits the generalizability of the findings to broader and more diverse populations. Therefore, future multi-center prospective studies with larger sample sizes are needed to validate and strengthen these conclusions.

In summary, our study presented novel evidence that T2DM, hypertension, and hyperlipidemia markedly elevate the risk of irAEs among cancer patients treated with ICIs. These findings highlighted the critical need for thorough pre-treatment evaluation and diligent monitoring of these comorbidities in ICIs therapy recipients. Future prospective clinical studies and mechanistic investigations into the immune-regulatory roles of T2DM, hypertension, and hyperlipidemia are necessary to enhance the safety of ICIs therapy.

## Data Availability

The original contributions presented in the study are included in the article/**Supplementary Material**. Further inquiries can be directed to the corresponding author/s.
