# Supplementary material for: Association of type 2 diabetes, hypertension, and hyperlipidemia with immune-related adverse events in patients undergoing immune checkpoint inhibitors therapy
Source: Front Immunol. 2025 Mar 7;16:1472197. doi: 10.3389/fimmu.2025.1472197 (PMC11925786; doi:10.3389/fimmu.2025.1472197)
Supplement: Supplementary file 1 [file Table1.docx]

**Table S1 Univariate logistic regression analysis of the association between comorbidities and irAE.**

| **Variables** | **OR[95%CI]** | ***P* - value** |
| --- | --- | --- |
| T2DM | 1.40 [1.13, 1.73] | 0.002** |
| Hypertension | 1.25 [1.04, 1.50] | 0.015* |
| Hyperlipidemia | 1.64 [1.04, 2.56] | 0.031* |

**Abbreviations:** irAEs, Immune-Related Adverse Events; T2DM, Type 2 Diabetes; OR, Odd Ratio. *P <0.05. **P <0.01.
